# The importance of content and face validity in instrument development: lessons learnt from service users when developing the Recovering Quality of Life measure (ReQoL)

**DOI:** 10.1007/s11136-018-1847-y

**Published:** 2018-04-19

**Authors:** Janice Connell, Jill Carlton, Andrew Grundy, Elizabeth Taylor Buck, Anju Devianee Keetharuth, Thomas Ricketts, Michael Barkham, Dan Robotham, Diana Rose, John Brazier

**Affiliations:** 1 School of Health and Related Research, University of Sheffield, Sheffield, UK; 2 School of Health Sciences, Medical School, University of Nottingham, Nottingham, UK; 3 Centre for Psychological Services Research, University of Sheffield, Sheffield, UK; 4 Department of Psychology, University of Sheffield, Sheffield, UK; 5 Institute of Psychiatry, Psychology & Neuroscience, King’s College London, London, UK; 6 The McPin Foundation, London, UK

**Keywords:** Quality of life, Recovery, Outcome measure, PROM, Validity, Service users, Qualitative

## Abstract

**Purpose:**

Service user involvement in instrument development is increasingly recognised as important, but is often not done and seldom reported. This has adverse implications for the content validity of a measure. The aim of this paper is to identify the types of items that service users felt were important to be included or excluded from a new Recovering Quality of Life measure for people with mental health difficulties.

**Methods:**

Potential items were presented to service users in face-to-face structured individual interviews and focus groups. The items were primarily taken or adapted from current measures and covered themes identified from earlier qualitative work as being important to quality of life. Content and thematic analysis was undertaken to identify the types of items which were either important or unacceptable to service users.

**Results:**

We identified five key themes of the types of items that service users found acceptable or unacceptable; the items should be relevant and meaningful, unambiguous, easy to answer particularly when distressed, do not cause further upset, and be non-judgemental. Importantly, this was from the perspective of the service user.

**Conclusions:**

This research has underlined the importance of service users’ views on the acceptability and validity of items for use in developing a new measure. Whether or not service users favoured an item was associated with their ability or intention to respond accurately and honestly to the item which will impact on the validity and sensitivity of the measure.

## Background

There has been an increasing commitment to Patient Reported Outcome Measures (PROMs) designed to measure day-to-day health improvements that matter most to service users [1, 2]. The methodological quality of PROMs has been determined by the extent to which they meet the recognised properties of measurement of reliability and validity [3–5]. A key property of validity is the extent to which a measure captures what it is intended to measure. In the absence of a gold standard measure, researchers have often used indirect methods for testing validity such as factor analysis, Rasch, or Item Response Theory (IRT). These are important in instrument development, but are insufficient on their own to fully establish the validity of an outcome measure [6]. It is also important to measure content validity; the extent to which the set of items comprehensively covers the different components of health to be measured [3] and face validity; whether the items of each domain are sensible, appropriate, and relevant to the people who use the measure on a day-to-day basis [7].

The content and face validity of many outcome measures currently in use are based on the judgements of researchers and health care professionals, with limited input from service users [8, 9]. However, what may be regarded as a good outcome by a clinician or researcher may differ from what is regarded as important to service users [10] and only service users can determine whether the measure captures these outcomes favourably [9]. Active input from service users in all the development stages of a measure can improve the acceptability, relevance, and the quality of the measure and related research [9, 11, 12].

In recent years, there has been a change in mental health policy from an emphasis on services that focus only on symptom reduction, towards those that take a more holistic and positive approach of service user-defined recovery and quality of life [13–15]. This growing movement towards positive mental health has given rise internationally to recovery-oriented mental health services [16–20]. It is of particular importance that PROMs used in these services reflect the key areas that service users consider relevant to recovery, rather than the traditional focus on symptom reduction [21]. Aspects of life that are considered important to ‘recovery’ have been shown to be consistent with those important to ‘quality of life’ [22, 23]. The themes service users consider important to quality of life: well-being, relationships and a sense of belonging, activity, self-perception, autonomy, hope, and physical health [24–26] are similar to those regarded as important to recovery in mental health: connectedness, hope, identity, meaning, and empowerment (CHIME) [27].

The new measure Recovering Quality of Life (ReQoL) in mental health was developed in four stages: (1) generation and subsequent shortlisting of candidate items; (2) testing face and content validity of shortlisted items; (3) psychometric evaluation; and (4) selection if the final items which involved combining the qualitative and quantitative evidence from stages 2 and 3. Importantly, service user opinion and input was utilised at all stages with a panel of six expert service users being involved in the selection of the shortlisted items from the pool of candidate items through to the decisions surrounding the inclusion of the final items. Psychometric testing involved over 4000 service users completing either a 60 or 40 item version of the measure before the selection of the final items. For details on all the stages involved in the development of the measure see Keetharuth et al. [28].

In this paper, we specifically report on the second of four stages which involved seeking the opinions of service users on a pool of items to help inform the selection of items which would go forward for psychometric testing. This builds on a systematic review of qualitative research and primary qualitative research involving service user interviews which identified the seven themes service users considered important to quality of life referred to above [24–26]. A pool of potential items (*n* = 1597) which best represented these seven domains was generated from current quality of life and recovery instruments and from service user interviews. The item set contained both positively (e.g. *I felt happy*) and negatively (e.g. *I felt sad*) worded items. These were subjected to initial sifting using a set of criteria adapted from those originally proposed by Streiner and Norman [4]. After consideration by clinicians, researchers, and service users who were members of the research study’s Scientific, Advisory, Stakeholder and Expert User Groups, the item set was reduced to 88 potential items for the new ‘Recovering Quality of Life’ measure [28]. The aim of this paper is to identify the key themes of face validity which should be considered when developing a quality of life instrument, using the ReQoL as an example. We also report on service users’ views on acceptability and validity of an item set.

## Methods

A qualitative study using face-to-face structured interviews and focus groups with service users was undertaken.

### Recruitment

There was a requirement that ReQoL be suitable for mental health service users over the age of 16. Therefore, adults (aged 19–79) and young adults (aged 16–18 years) from National Health Service (NHS) mental health services and a local charity were invited to participate. In order to obtain views from across the spectrum of mental health service users, broad inclusion criteria were applied; the only exclusion concerned people experiencing acute episodes of their mental health condition, those not well enough to take part, and those who could not speak English or give informed consent. This allowed for maximum variation of mental health problems, severity of problems, and current service contact.

Adult participants were recruited from four UK NHS Trusts providing mental health services and a UK charity in the north of the country. Two trusts were located in the south of England and two in the north. Recruitment of young adults (aged 16–18) took place from two further NHS trusts based in the Midlands and the North of England. Recruitment was undertaken on our behalf by healthcare staff and clinical studies officers within the individual trusts. Recruitment procedures followed what was usual and practical for each individual Trust. This variously involved healthcare staff approaching service users on inpatient wards, when attending therapy sessions, those who had previously agreed to be approached for research purposes, attendees at a Recovery College and at a Rehabilitation and Recovery Centre, and members of established PPI (Public and Patient Involvement) groups. At the time of the interview, demographic, care service, and diagnostic information was taken. In order to obtain a representative and diverse sample, this information was used to identify recruitment gaps and recruitment personnel were subsequently informed and asked to target these groups.

### Participants

A total of 59 adult service users took part: 40 participated in individual interviews; 11 attended two focus groups of seven and four participants, respectively; and four interviews took place with two participants together. A total of 17 young adults participated: 15 participated in individual interviews and two participants chose to be interviewed together. Interviews lasted between 20 min and 1 h 40 min. For information on the sample demographics please refer to Table 1.

**Table 1:** Characteristics of participants

	Adults		Young adults	
	*N*	%	*N*	%
Gender				
Male^a^	22	37.3	5	29.4
Female	37	62.7	12	70.6
Age				
16–18	0	0	17	100
19–29	12	20.7	0	0
30–39	15	24.1	0	0
40–49	12	20.7	0	0
50–59	10	17.2	0	0
60–69	6	10.3	0	0
70+	1	1.7	0	0
Not indicated	3	6.8	0	0
Ethnicity				
White British	46	77.6	15	88.2
Black/Black British	6	10.3	0	0
Asian/Asian British	2	3.4	0	0
Mixed/multiple ethnic group	2	3.4	2	11.8
Other ethnic group	3	5.2	0	0
Employment/activity				
Employed	16	25.9	2	11.8
Student	2	3.4	15	88.2
Retired	4	6.9	0	0
House-person	2	3.4	0	0
Not in employment	25	43.1	0	0
Other	8	13.8	0	0
Not indicated	2	3.4	0	0
Diagnosis/own view^b^				
Schizophrenia/psychosis	26	44.8	0	0
Depression	27	46.6	6	32.3
Bipolar	8	13.8	0	0
Anxiety disorders	21	24.4	4	23.5
Eating disorder	2	3.4	2	11.8
Personality disorder	4	6.9	0	0
Other	4	6.8	0	0
Not indicated	2	3.4	5	29.4
Current care^c^				
None	3	5.2	0	0
General practitioner	6	10.3	1	5.9
Improving access to psychological therapy	8	13.8	0	0
Community mental health team	29	50.0	0	0
Child and adolescent mental health services (CAMHS)			14	82.3
CAMHS inpatient			2	11.8
Adult inpatient	8	13.8	0	0
Voluntary sector	3	3.4	0	0
Not indicated	2	3.4	0	0

^a^One participant identified as being transgender

^b^Participants provided a diagnosis and their own opinion of their problem—data here are a combination of the two with priority given to the diagnosis where provided. Some participants provided more than one diagnosis/opinion of their presenting problem

^c^Some participants indicated that they were receiving current care from more than one service provider

### Interviews

Interviews and focus groups were conducted between September 2014 and June 2015. Interviews and focus groups were undertaken at NHS sites familiar to the participants apart from one which was on university premises. Participants were provided with an information sheet prior to the interviews. Written consent was obtained at the time of the interview prior to any data collection. Participants were asked to complete a short demographics form indicating their gender, age, ethnicity, employment, education level, mental health diagnosis, and their own perception of their mental health problem (which may or may not be the same as the diagnosis). Interviews and focus groups with adult service users were conducted by three experienced qualitative researchers, one of whom is a service user (JCo, JCa, AG). Interviews with young adults were undertaken by an experienced qualitative researcher and a clinician who specialises in child and adolescent mental health (ETB). All participants were given a £20 shopping voucher in recognition of their time.

The interview process was the same for both adult and young adult service users. The items were grouped by domain, ordered, and presented one domain at a time. To reduce fatigue effect, the items were presented in a different order by starting with a different domain for each subsequent interview. To help establish the validity of each potential item, participants were asked whether it was meaningful and relevant to their quality of life; whether it was clear, understandable and easy to answer; and the reason they either liked or disliked an item, or preferred it to another. They were also asked for their preferred item within a group or pair of items thought by them to relate to a similar concept (e.g. between ‘I felt relaxed’ and ‘I felt calm’). Alternative wordings to items were elicited if participants thought an item was important to their quality of life but unclear or difficult to answer.

An iterative approach was undertaken with the interviews. Adult participants were initially presented with a set of 88 potential items. At approximately the halfway stage after interviews had been undertaken at two of the trusts, 12 items (which were primarily a reformulation of existing questions) were added as a result of the feedback provided by those participants who had been interviewed. This increased the item set to 100 potential items for the remaining interviews. As a result of the findings from the adult participants, a meeting was held with scientific and user group members and decisions were made to remove some items from the set and to change others. This reduced the number of items to 61. Young adults were presented with this reduced item set. Due to the large size of the item set, not all participants provided their views on all items. The average number of items commented on by the adult group was 68; the lowest number was 5 by a person who was unwell at the time and the interview was terminated early, and also 19 items by a focus group of 7 people. The maximum number of responses was all 100 items. The average number of items commented on by the young adults was 34; the lowest number was 21 and the highest was all 61 items. The comfort and enthusiasm of the interviewee to continue with the interview was considered at all times. The majority of the interviews were recorded and these recordings were transcribed. Notes were taken for the three adult interviewees who preferred their interview not be recorded. All identifying information was removed from the transcripts and notes prior to analysis.

### Analysis

A pragmatic approach was taken in the analysis of the interview data. From the transcripts, the comments made by each participant for each item were charted into a spreadsheet framework with items across the horizontal axis and participants on the vertical axis. A traffic light system was used to highlight negative (red), positive (green), and neutral or ambiguous comments (orange). Those items with relatively high levels of acceptability and unacceptability were identified. A thematic analysis of the comments was undertaken to establish the underlying reasons for the popularity, or lack of popularity, of the items. This information was used as a starting point for discussion with the scientific, advisory, and expert user groups to establish whether or not an item should remain as a potential item in the ReQoL measure. In the final stages of the development of the measure, this information was used in conjunction with other evidences such as the psychometrics and feedback from clinicians [28] to make a decision on the final items.

## Results

Analysis required that the items included in the measure should be relevant and meaningful, be unambiguous and easy to answer when feeling distressed, did not cause further distress, and were non-judgemental. Importantly, this was from the perspective of the service user. Interviewee quotes relating to these issues and the respective items can be found in Table 2.

**Table 2:** Interview participant quotes about potential items for inclusion in the ReQoL measure

Quote no.	Item	Quote (participant) [decision]
Item relevance		
1	I felt able to trust others	“A really good question—if you’re feeling not so good or a little bit paranoid or whatever then you know the trust definitely goes down. And I just think that’s important as well because I think if you don’t feel like you can trust other people then you’re certainly not going to feel very happy” (AI24). [Retained]
2	I felt confident in myself	“You need confidence to be able to value yourself—when you have got no confidence and you are down you don’t value nothing” (AF3) [Retained]
3	I felt loved	“I think to feel loved might be a luxury—but to feel cared for erm might be sufficient. I mean I don’t suppose the health service can erm prescribe or give love but they can provide care” (AI6) Omitted—item ‘I thought people cared about me’ [Retained]
4	I could do all the things I wanted to do	“I don’t think (this question) is very valid—I can’t do all the things I want to do—swim with dolphins, it just isn’t going to happen (laughs). No” (AI12) [Changed to ‘I could do the things I wanted to do’]
5	I felt tired and worn out	“… feeling tired and worn out, can be like fatigue or lots of different things […] a lot of teenagers feel like tired and worn out all the time because it’s just kind of how we are, sort of thing, but like if like everything is an effort, like even like brushing your hair is an effort, that’s sort of like different to feeling a bit tired (YPI14) [Omitted]
6	I feel confused about who I am	“I think that you’re going to get that with a lot of people really and I think it just like develops, like as you get older you tend to get less-, […] because at that time you’ve got lots of like you’ve got a lot of hormone changes and imbalances in your body and it’s just you get mood swings all the time and it’s just like you’re going through lots of different things and it can just be…” (YPI3) [Omitted]
Item ease of response		
7	I felt discriminated against	“I mean I would think if I was answering this and I would look back I would think ‘I don’t know’, I think it’s a hard thing to say that someone has discriminated against you.” (AF5) [Omitted]
8	I thought people did not want to know me	“I don’t know what other people think. ‘Didn’t want to know me’, what do you mean by that? … It’s very vague isn’t it, sort of verging on a paranoid thought isn’t it.” (AI34) [Omitted]
9	I neglected myself	“I think that when I have been neglecting myself I wouldn’t have known so like maybe I hadn’t showered for like three weeks but I probably wouldn’t realise that I was neglecting myself.” (I31) [Omitted]
10	I was thinking clearly	“At the time you think you are thinking clearly, especially if you are in an episode of psychosis what is in your head is very real so I think people could probably answer yes to that one.” [Omitted]
Ambiguous items		
11	I felt guilty	“There are two types there is a good one and bad one, it is good to have guilt because it shows that you are a decent person … it is a good thing, it is another thing that shows you are getting better cos when I was committing my crimes, I didn’t think that I had done owt wrong because I weren’t well like, and when I got well the guilt kicked in, so I think a guilty sign is when you are getting well—but to have too much it can ruin your life can’t it?” (AF3) [Omitted]
12	I felt everything was under control	“Coping is getting through it, just getting through it, but controlling is being in charge […] the control may be too much for some people. Do you know what I mean?” (YPI2) [Changed to ‘I felt in control of my life’]
13	I felt hopeless	“Erm I think that you could say I felt hopeless at erm at like carrying out a task or…”(I: so for you hopeless there means useless?) “Yes”. (I: you would understand that to mean, I felt useless?) “Yes” (I: Not I felt without hope?) “No I don’t think so [...] I would never think about it like that.” (YPI1) [Omitted]
Distressing or sensitive items		
14	I had thoughts about killing myself	“I don’t like ‘killing myself’ I don’t like that expression, I will be honest with you, maybe ‘have you had thoughts about self-harm’, could it be worded another way? I would agree it is important to ask because lots and lots of people do have suicidal thoughts.” (AI15) [Omitted—item ‘: I thought my life was not worth living’ retained]
15	I detested myself	“I think I would be knocked back by it (*this question*) I think, I would be like ‘oh do I’, you know and then it’s not, it brings on feelings like ‘well yes I am this and I am that’ … I think it might be too strong a word because it might bring back awkward feelings.” (AI26) “’Detest—it might be a bit embarrassing to say and dislike is a bit of an easier one to swallow.” (AI4) [Omitted]
16	I had problems with self-care, washing or dressing	“I wouldn’t like to answer that, it’s making me feel a bit ashamed that I can’t take care of myself.” (AI36) [Omitted]
17	I have threatened or intimidated another person	“That’s quite extreme isn’t it so I wouldn’t like to answer that question.” (AI38) [Omitted]
18	I felt full of life	“I think it’s that thing where people might think ‘well I wouldn’t be here if I was full of life and happy’ .... I think it’s got to have an air of realism about it. For the person who’s reading it to think that it’s a good reflection of how they’re feeling or you know you’ve understood their situation. I just think that they’re a bit too wahooo, here’s your party banner and your balloon kind of thing.” (AI24).[Omitted]
Judgemental items		
19	I felt I made a contribution/I was able to do things that helped others	“Is that a sign of being well, you could just be a selfish person? For some people it wouldn’t be part of their life to do something to help other people so there is no relevance. My charity work helps me and helps others—but that is more to do with my work ethic than wanting to help others. If I saw something like that in a questionnaire when I was feeling very poorly it would just make me feel worse because to me that looks like a judgement. You could be making a contribution but it’s actually making you more ill so is it a positive thing or is it actually a negative thing?” (AF1) [Omitted]
20	I lived as independently as I would like to	“I’m not sure. I’m not sure if living independently is an imitation of erm good mental health … and I think there’s an implication with being independent that you’re doing alright and if you’re not, you’re not.” (AI6) [Omitted]

### Item relevance

There were some items which were universally liked and had few objections. What these items had in common was that the service users could relate to the item as being something they experienced regularly. As a result, responses to these questions did not require much thought and were considered easy to answer by the vast majority of participants. Items which fell into this category were ‘I had difficulty getting to sleep or staying asleep’; ‘My health limited day to day activities’; ‘I felt able to trust others’ (Quote 1); ‘I felt anxious’ and ‘I felt confident in myself’ (Quote 2). These items were considered to be particularly relevant because of the further impact these experiences or feelings had on other aspects of their quality of life, for example, if you were unable to trust people, then you would never be happy, and how self-confidence impacted on self-worth.

Some items were described by the service users as being irrelevant or meaningless, either to their own mental health problems or to quality of life. For example, when considering the item ‘I felt accepted as who I am’, service users felt that is was more important to quality of life that they accepted themselves rather than be accepted by others. It was felt that it was not necessary to ‘feel loved’ (Quote 3) for a good quality of life, and was perhaps a bit of a luxury, but feeling ‘cared for’ was important and less specific to a certain type of relationship. It was also felt impractical and unachievable to have ‘everything under control’ or be able to ‘do all the things I wanted to do’ (Quote 4).

There were objections to some items because they were thought to be too general and not specific to mental health difficulties. Examples included ‘I avoided things I needed to do’—it was felt that there may be very good reasons to avoid doing things you did not want to do and doing so could enhance your mental health; ‘I felt irritated’ which was felt to be a normal reaction and not necessarily linked to mental health; and ‘I felt tired and worn out’ (Quote 5) which could be due to physical health as well as mental health. A few of the young adults thought that being ‘confused about who I am’ (Quote 6) was a natural thing for individuals in their age group.

### Ease of response

Some items were considered difficult to answer by some participants because they were too abstract, thus requiring too much thought. Whilst under normal circumstances this would not be a problem, it was something they felt could be particularly difficult when in a distressed state upon first accessing mental health services. This particularly applied to items where they thought they were required to consider the thoughts of others, e.g. ‘I thought people did not understand me’; ‘I felt discriminated against’ (Quote 7); ‘I felt accepted as who I am’; and ‘I thought people did not want to know me’ (Quote 8). Rather than considering whether they thought people understood them (the intention of the item), some would try to think about eg whether people did understand them.

There were some items that the service users thought may be difficult to provide an honest answer to because of their own low level of self-awareness whilst ill. It was sometimes only in retrospect that they may realise they had ‘neglected themselves’ (Quote 9) or were not ‘thinking clearly’ (Quote 10).

### Item ambiguity

Some items could be interpreted in more than one way, for example, whether an item related to physical or emotional health. ‘I was in pain’ was considered by some to be about emotional rather than physical pain, and there was some ambiguity whether ‘I had problems with self-care, washing or dressing’ was related to emotional or physical problems, or both. There were also items that were intended to be negative (i.e. indicative of a poor quality of life) but in some circumstances could be regarded as positive. For the item ‘I felt guilty’ (Quote 11), a couple of the interviewees indicated that this could be a positive change when they had done something regrettable whilst ill. Similarly, items that were intended to be positive could be interpreted negatively. For example, ‘thinking clearly’ could be due to a total lack of emotion and doing something ‘bad’ could be ‘enjoyable or rewarding’. For young adults, the item ‘I felt everything was under control’ (Quote 12) was not necessarily indicative of good quality of life as it meant being in charge, and the item ‘I was able to cope with everyday life’ was preferred as it indicated they were dealing with it without having to take over control. Young people also thought the item ‘I felt hopeless’ (Quote 13) had two interpretations of ‘lack of hope’ and ‘feeling useless’.

### Distressing or sensitive items

One of the most common reasons given for objecting to an item, and having the view that it should not be included in a quality of life measure, was that it would cause upset. Some items were considered to be *too* negative. These were often related to suicidal thought and intent. It was the extreme negative (and direct) wording within items that participants found distressing, such as ‘I had thoughts of killing myself’ (Quote 14) and ‘I thought I would be better off dead’. The wording was described as being ‘upsetting’, ‘harsh’, and ‘too strong’ and could actually provoke suicidal thoughts. However, the majority of participants thought that suicidal intent was an important indicator of quality of life but expressed a preference for items with a more indirect, sensitive approach. The items ‘I did not care about my own life’ and ‘I thought my life was not worth living’ were preferred. Other items considered to be too extreme by some participants, and thus described as upsetting, related to feelings about the self. The items ‘I felt humiliated or shamed by other people’; ‘I felt useless’; ‘I felt shame’; ‘I felt stupid’; and ‘I detested myself’ (Quote 15) were described as ‘too personal’, ‘embarrassing’, ‘not nice’, and ‘traumatising’. One participant stated that such a negative question about the self would make ‘the voices’ more prominent. For items relating to the self, the positive items (e.g. ‘I felt confident in myself’; ‘I felt ok about myself’) were preferential. Of the negative items, again a gentler approach was favoured, e.g. ‘I disliked myself’ rather than ‘I detested myself’.

Due to its sensitive nature, a number of people responded that they would not like to admit to certain things and therefore would either not respond to the item or would answer dishonestly. The reasons given were that they would find it embarrassing [‘I felt humiliated or shamed by other people’; ‘I had problems with self-care, washing or dressing’ (Quote 16)] and had concerns surrounding the consequences of disclosure [‘I had thoughts about killing myself’; ‘I have threatened or intimidated another person’ (Quote 17)].

Some items were felt to be insensitive because they were *too* positive, in particular the items ‘I felt full of life’ (Quote 18), ‘I felt I could bounce back from my problems’, and, to a lesser extent, ‘I felt happy’. Participants felt these items to be unrealistic, as they thought they were never likely to feel this way even when they were well. Such items were described as ‘patronising’ and ‘daft’, particularly if asked when they were accessing a mental health service for the first time when they would be feeling particularly distressed.

### Judgemental items

Some positively worded items were thought to be too judgemental and reflected an opposing value system. This particularly applied to those items that were related to doing ‘good’ things ‘I was able to do things that helped others’ (Quote 19); ‘I felt I made a contribution’ (Quote 19); ‘I did things that I found worthwhile’; and ‘I felt useful’. It was felt by some participants that helping others was not necessary for quality of life and that people with mental health problems were not necessarily in a position to be able to help others or to do things that were worthwhile, and doing such activities could make them feel worse. Furthermore, participants noted that if the items were answered truthfully (that they did not do things that helped others) this may result in feelings of guilt. The concept of ‘independence’ was also thought to be judgemental because of the assumption that independence was something to be valued (Quote 20).

## Discussion

Recent guidance advocates clear and transparent reporting of measure development and assessment of instrument properties [2]. Reliability and psychometric properties of instruments are frequently reported, but a key stage of the development of any measure is that of content and face validity. The assessment of content and face validity and the acceptability of items by those people who will ultimately use the measure can only be achieved through qualitative work with service users [6]. This paper demonstrates the importance of considering service users’ views on potential items. Service users provided their views on potential items to be considered in the ReQoL measure. In summary, they expressed concerns about items that were sensitive and could potentially cause distress; judgemental of what was a good outcome; not relevant to their mental health and quality of life; ambiguous in their interpretation; and difficult to answer either because they were too vague or abstract. The service users indicated that the potential consequences of including such items would be that they would not respond to the item accurately, truthfully, or at all, which in turn would have a detrimental effect on the validity of the measure. This is especially concerning as most of the items tested were from, or adapted from, measures currently in use.

Our findings are consistent with those of Crawford et al. [29] who retrospectively sought the views of service users about the appropriateness of commonly used measures in mental health. One of the primary concerns expressed in their study was where an item was judgemental in its criteria of ‘good’ outcome: for example, the view that people who got on well with family members had better social functioning or quality of life. The items susceptible to this criticism in our set of potential items related to the concepts of ‘independence’, ‘helping others’, and ‘contributing to society’ by doing things that were useful or worthwhile.

Crawford et al. [29] also recommended that a measure should not comprise a long list of questions about difficulties associated with mental ill health but rather it should consist of positive as well as negative items. We did find that some items could be considered too extreme in their negative or positive nature. Those considered too negative by our participants predominantly related to suicidal thoughts and those considered to be too positive related to higher levels of well-being (happiness, joy, and fun). This presented some concerns for the ReQoL team as it was considered important that a valid quality of life measure should cover the full spectrum of mental health from the worst to the best achievable [30]. Additionally the polar aspects of the stages of recovery should also be represented; from a profound sense of loss and hopelessness to living a full and meaningful life [31]. So, whilst service user concerns were acknowledged, a decision was made to retain to the next stage of development those items which were considered the least extreme and least objectionable to service users but still reflected a sense of hopelessness, and positive well-being. A similar dilemma occurred with items relating to a negative sense of self. The theory of stages of recovery starts with a notion of ‘negative identity’ through to a ‘positive sense of self’ [31]. There were no objections related to items having an overly positive sense of self but there were some that caused upset because they were unduly negative (e.g. I detested myself, I felt stupid). Again, in order to cover a broad spectrum of ill health, those that were negative but considered less insensitive were retained.

Some participants objected to items they thought could easily be applied to any member of the general public rather than being specific to those with mental health problems (feeling irritated, avoiding things, tiredness). After discussion, it was decided that this did not necessarily warrant omission of these items as the measure is designed for people at different stages of their recovery.

To maximise the acceptability, validity, and reliability, the findings from this research informed the development of the ReQoL measure at every stage. As a result of the feedback from service users, some items were omitted, whilst others were reworded. After psychometric assessment of item performance, the findings were again used to inform the final item selection for the ReQoL measure alongside clinician input [28].

For an instrument to measure the concepts most significant and relevant to a service user’s difficulties, it is important that those providing feedback are representative of the target population [6]. This was considered as far as possible and people with a wide variety of diagnoses, with different severity levels and from inpatient, outpatient, and recovery services were recruited and interviewed. However, it should be noted that a small proportion of those with the greater severity of problems at the time of the interview were less able, or motivated, to provide a comprehensive response. They were happy to indicate whether or not they liked an item but did not always articulate why. A greater depth of response was given by those who had milder conditions or those who were relatively well at the time of the interview, though they were able to reflect back on when they were less well. Due to time constraints, we were unable to interview people from primary care services.

Whilst care was taken at the initial selection of items using an established list of criteria for choosing the best items, this stage made it clear that the responses of the service users were not always those expected by the researchers. This further reinforces the fact that a key component of measure development is that of content validity to the target population. Here we have shown that certain questions can be considered irrelevant, judgemental, distressing, ambiguous, or difficult to answer and care should be taken when developing a measure to be cognisant of this. However, the items that one population of service users find objectionable is likely to differ from another. This paper demonstrates that the involvement of service users within this important methodological stage is paramount to the development of a reliable and valid measure that will be acceptable to those who complete it. Further independent research on service users’ acceptance of the measure and items compared with other quality of life measures would be welcomed.

## Data Availability

The data that support the findings of this study are available from the corresponding author. However, restrictions apply to the availability of these transcripts as the participants only consented to their data being accessible to the researchers involved in the project.
